# The prevalence of internet addiction and its association with quality of life among inflight security officers based on a national survey: a network analysis perspective

**DOI:** 10.1007/s00406-025-02030-y

**Published:** 2025-06-09

**Authors:** Zhen Gui, He-Li Sun, Yuan Feng, Qinge Zhang, Zhaohui Su, Teris Cheung, Gabor S. Ungvari, Erliang Zhang, Minzhi Chen, Jie Zhang, Lin Zhang, Bin Ren, Qingqing Jin, Chee H. Ng, Mi Xiang, Yu-Tao Xiang

**Affiliations:** 1 Unit of Psychiatry, Department of Public Health and Medicinal Administration, and Institute of Translational Medicine, Faculty of Health Sciences, University of Macau, Macao SAR, China; 2 Centre for Cognitive and Brain Sciences, University of Macau, 1/F, Building E12, Macao SAR, China; 3 Beijing Key Laboratory of Mental Disorders, National Clinical Research Center for Mental Disorders & National Center for Mental Disorders, Beijing Anding Hospital, Capital Medical University, Beijing, China; 4 School of Public Health, Southeast University, Nanjing, China; 5 School of Nursing, Hong Kong Polytechnic University, Hong Kong SAR, China; 6 Section of Psychiatry, University of Notre Dame Australia, Fremantle, Australia; 7 Division of Psychiatry, School of Medicine, University of Western Australia, Perth, Australia; 8 School of Public Health, Shanghai Jiao Tong University, Shanghai, China; 9 CAAC East China Aviation Personnel Medical Appraisal Center, Shanghai, 200336 China; 10 Department of Psychiatry, The Melbourne Clinic and St Vincent’s Hospital, University of Melbourne, Richmond, VIC Australia; 11 Hainan Branch, Shanghai Children’s Medical Center, School of Medicine, Shanghai Jiao Tong University, Sanya, China

**Keywords:** Internet addiction, Quality of life, Inflight security officer, Network analysis

## Abstract

**Background:**

Given the heavy responsibilities placed on inflight security officers (IFSO) to ensure passenger safety and eliminate inflight hazards, they often turn to Internet use to cope with their work pressure. This study examined the prevalence of internet addiction (IA) among IFSO in China, and its associated factors, relationship with quality of life (QOL), and network structure.

**Methods:**

This was a cross-sectional study based on a national survey. Expected influence (EI) was used to identify the most central nodes within the network model.

**Results:**

Among 3,475 IFSO included in this study across 10 airlines, the prevalence of IA (IAT-20 total score of ≥ 50) was 13.1% (n = 454; 95% CI: 11.9–14.2%). Further, there was an association between lower annual income, more severe depressive symptoms and anxiety symptoms with IA among IFSOs. Network analysis found that “Job performance or productivity suffer because of the Internet” (IAT8) was the most central symptom with the highest EI value, followed by “Preoccupation with the Internet” (IAT15) and “Depressed/moody/nervous only while being offline” (IAT20). Moreover, “Sleep loss due to late-night logins” (IAT14) and “Spend more time online over going out with others” (IAT19) had the most negative associations with QOL, while “Form new relationships with online users” (IAT4) showed the strongest positive correlation with QOL.

**Conclusion:**

IA was common among IFSO. To reduce the adverse impact of IA among IFSO, appropriate interventions targeting central symptoms and those closely associated with QOL found in the network models should be developed.

**Supplementary Information:**

The online version contains supplementary material available at 10.1007/s00406-025-02030-y.

## Introduction

In China alone, the air industry reported that passenger aircrafts completed 4.92 million flights, totaling 12.21 million flight hours, and transported 620 million passengers, representing year-on-year increases of 94.5%, 91.8%, and 146.1%, respectively in 2023 [1]. Given that aviation security and safety has become a high priority worldwide, the Chinese government has therefore highlighted the important role of inflight security officers (IFSO) [2].

IFSO refer to aircrew who are deployed on aircraft to perform air safety and security tasks authorized by the government of the operator's country of registration. Their primary work responsibility is to protect the aircraft and their occupants from unlawful interference [3], ensure the safety of passengers and eliminate safety hazards. Furthermore, they need to have specialized work skills, strong psychological qualities and keen ability to identify risks [4]. Although the work environment of IFSO can be stressful and complex, the routine work content is usually monotonous, which makes IFSO prone to work fatigue. Due to frequent time zone changes, long-distance flights and shift work patterns with a lack of regular work and rest schedules, IFSO usually experience high levels of work intensity, pressure and fatigue [5]. As a result, they often turn to Internet resources for relaxation and communication [6].

Over the past decades, excessive use of the Internet has become a common problem [7, 8], and the risk of Internet addiction (IA) has been increasing across many populations [9]. IA refers to a range of problems due to a lack of control over Internet use, withdrawal symptoms and tolerance characteristics [10, 11]. Notably, IA is not classified as a psychiatric diagnosis in the Diagnostic and Statistical Manual of Mental Disorders, Fifth Edition (DSM-5) [12], whereas Internet Gaming Disorder (IGD) is listed in the International Classification of Diseases 11 th Revision (ICD-11) [13], and may also be referred to as “Gaming Addiction,” or “Internet Use Disorder,” or “Internet Addiction” [14]. A meta-analysis of data from 31 countries worldwide revealed that the prevalence of IA varied widely ranging from 0.5% to 40%, with the overall prevalence of 7.02% (95% confidence interval (CI): 6.09–8.08%) [15]. In addition, the risk factors of IA commonly reported included young age, male gender, low self-esteem, loneliness and social isolation [16, 17]. Due to high work pressure and irregular schedules, the Internet is often the main source of social interaction, leisure activities, and stress relief for IFSO [6, 18]. However, IA is also related to a host of negative psychological health problems (such as depression and harmful alcohol use), poor cognitive performance, increased hostility/aggression, interpersonal difficulties and decreased quality of life (QOL) [19–21]. From an aetiological view point, IA is closely associated with functional and structural changes in the prefrontal cortex and striatum, which may impair impulse control [22, 23]. Furthermore, extensive use of the Internet and digital devices, family dysfunction, and social pressures are factors that can increase the risk of IA [23, 24]. Since IA involves biopsychosocial factors, effective intervention to prevent and treat IA requires a multidisciplinary approach such as cognitive behavioral therapy (CBT) [25], pharmacological treatment (such as selective serotonin reuptake inhibitors, SSRIs) [26], digital literacy education, and lifestyle modifications [27]. To facilitate the evaluation of IA in both research and clinical practice, various questionnaires to measure IA have been developed. Of them, the Internet Addiction Test (IAT) is the most commonly used and reliable instrument globally [28].

Network analysis is a data-driven statistical method for assessing and visualizing the structure and interconnections of psychiatric symptoms [29, 30]. Compared to traditional statistical methods, network analysis identifies central symptoms and elucidates the complex relationships between symptoms within a network model [31, 32]. Targeting central symptoms can improve treatment efficacy by addressing the mechanisms involved in the aetiology and maintenance of psychiatric problems [29]. Network analysis has been widely used among psychiatric disorders, including major psychiatric disorders [33], anxiety [34], suicidality [35], problematic smartphone use symptoms [36] and IA [37]. For example, a network analysis of major depressive disorder (MDD) patients revealed that the “Preoccupation with the Internet” was the most influential (central) symptom in IA [38]. However, to date, most network analysis researches of IA mainly paid close attention to children and adolescents as well as those with psychiatric problems [33, 39–42]. Due to the different characteristics of such study samples, previous results could not be generalized to other groups such as IFSO in which there is a paucity of research.

To address this research gap, this study explored the prevalence and associated factors of IA, and its relationship with QOL and network structure among IFSO in China, using the nationally representative data from the Civil Aviation Health Cohort of China (CAHCC). As QOL is a widely used indicator of health outcomes, understanding its associations with IA symptoms is crucial for mitigating the negative impacts of IA on QOL [43].

## Methods

### Study design and participants

Following previous studies [44, 45], a convenience sampling method was adopted for this study due to logistical reasons. A questionnaire link was sent to all IFSO working in the participating airline companies through the Short Message Service (SMS) function of the Questionnaire Star online questionnaire platform. Recipients could access the survey by clicking the provided link and then completing it online. In addition, the baseline assessment data was collected from December 2022 to March 2023 in a national survey, the study included IFSO from 10 airline companies nationwide in China. To achieve a satisfactory response rate, a supervision group consisting of team leaders of IFSO from each participating company was set up to monitor the response rate daily. To increase the participation rate of IFSO, the team leaders in the participating airline company encouraged the IFSO to complete the assessment during the study period. The participants were: 1) working as IFSO during the survey period; 2) older than 18 years; 3) able to provide electronic written informed consent. Those with a history of psychiatric disorders including substance and alcohol dependence were excluded. In addition, The Ethics Committee of Civil Aviation Shanghai Hospital has approved this research protocol.

### Measures

The sociodemographic information including age, gender, education level, marital status, annual income level, and working duration were collected. The validated Chinese version of the self-reported 20-item Internet Addiction Test (IAT-20) was used to evaluate the IA symptoms over the past month [46, 47]. The IAT-20 contains 20 items across six domains: Excessive use, Salience, Neglect work, Anticipation, Lack of control, and Neglect social life. Each item is rated on a 5-point Likert scale from"1"(rarely) to"5"(always), with the total score ranging from 20 to 100 points. The IAT has been validated in Chinese populations [35].Following previous studies [33, 48], participants with a IAT total score of ≥ 50 was defined as “having Internet addiction” [28, 49, 50]. Such dichotomization could enable the application of binary logistic regression to examine the associated factors of IA in a clinically relevant manner.

The validated Chinese version of the nine-item Patient Health Questionnaire (PHQ-9) was used to assess the severity of depressive symptoms [51, 52]. The PHQ-9 consists of 9 items, with each item scoring from 0 to 3 (from “not at all” to “nearly every day”) and the total score ranging from 0 to 27. A higher total score indicates more severe depressive symptoms [53]. The validated Chinese version of the seven-item Generalized Anxiety Disorder scale (GAD-7) was used to assess anxiety symptoms. The GAD-7 consists of seven items, with each scoring from 0 (“not at all”) to 3 (“nearly every day”) [54, 55]. The GAD-7 total score ranges from 0 to 21, with a higher total score indicating the more severe anxiety symptoms [56].

Global quality of life (QOL) was measured using the Chinese version of the World Health Organization Quality of Life-brief version (WHOQOL-BREF) [57]. Specifically, QoL was measured as the sum of the first two items: “How would you rate your quality of life?” and “How satisfied are you with your health?”. The validity of such QoL measure has been well established and widely used across Chinese populations [58, 59]. Each item is scored from 1 (extremely dissatisfied) to 5 (extremely satisfied) and the total score ranges from 2 to 10 points. [60].

### Statistical analysis

#### Univariate and multivariate analyses

In this study, univariate and multivariate analyses were used the SPSS 27.0 (SPSS Inc., Chicago, Illinois, USA). The characteristic data between subgroups with and without IA symptoms were compared using independent sample t-tests for continuous variables and Chi-square tests for categorical variables. After controlling for variables with significant group differences in univariate analyses, analysis of covariance (ANCOVA) was used to compare QOL between IFSO with and those without IA. Binary logistic regression analysis was used to test the independent associated factors of IA, and variables with significant differences between groups in the univariate analysis were entered as independent variables. P < 0.05 was set as statistically significant (two-tailed).

#### Network analysis

R program (Version 4.3.1) [61] was used for the network analysis to examine the interconnections between IA symptoms in IFSO. Network analysis is an innovative statistical method that visualizes the relationships between different symptoms in a network model, where node represents IA symptoms, and the edge between the two nodes represents the correlation between both symptoms. A thicker edge signifies a stronger correlations, while the color denotes the direction: green edge indicating positive correlations, while red edge indicating a negative correlation [62].

The *estimateNetwork* function in the *“networktools”* package (Version 1.5.1) [63] with the *“EBICglasso”* method was used to estimate the network model of IA symptoms. The *“qgraph”* package (Version 1.9.8) [62] and the *“ggplot2”* package (Version 3.4.4) [64] were used for network visualization and optimizing network visualization, respectively. To accurately evaluate the association between different IA symptoms in the network model, we used the Extended Bayesian Information Criterion (EBIC) [62] and Graphical Gaussian Model (GGM) with the least absolute shrinkage and selection operator (LASSO) [62] to simplify the network model and improve its interpretability [65].

In the network model where individual symptoms are treated as nodes, a greater centrality index of nodes indicates a higher importance of symptoms within the network model. Following previous research [66, 67], to identify the most central (influential) symptoms in the network model, we calculated the centrality measure to quantify the importance of network nodes. Among the various centrality indices, expected influence (EI) was adopted because it could indicate both positive and negative edge weights, providing a more comprehensive measure of the overall impact of symptoms. The EI centrality was calculated by summarizing the sum of positive and negative edges connected to a specific node, which could identify the most influential nodes within the network model [68]. In contrast, other measures of centrality, such as strength centrality, could only calculate the absolute value of the connection but ignore the directional effects of symptoms. A larger EI value indicates the greater the node's influence within the network model. Since the central (influential) symptoms are more closely related to other symptoms in the network model, treatment that target the most central symptoms could be more effective in improving the psychiatric disorder or syndrome [69].

Moreover, *“mgm”* (Version 1.2–14) [70] was used to calculate the predictability for IA symptoms, thereby quantify the degree to which each symptom could be explained by its direct connections within the network. In addition, the *flow* function in the *“qgraph”* package (Version 1.9.8) [62] was used to identify specific IA symptoms that were directly and indirectly related to QOL.

R-package *“bootnet”* (Version 1.5.6) [71] was used to evaluate the EI centrality stability and edge accuracy. Bootstring difference tests were used to evaluate the robustness of edges and nodes EI in the network model, in which correlation stability coefficient (CS coefficient) was used to evaluate the robustness of EI centrality. When CS coefficient was greater than 0.25, the results of the network model were stable and reliable. The network with CS-coefficient greater than 0.5 had better robustness [72]. The accuracy of the edge was estimated using the bootstrap 95% confidence interval (CI), and the narrower the estimated 95%CI, the higher the reliability of the network [72]. When 1,000-bootstrap 95% confidence interval (CI) value range did not contain zero, the difference between two nodes or two edges was significant.

## Results

### Study sample and descriptive statistics

Of a total of 3,755 in-service IFSO from 10 airlines invited to participate in the survey, 3,475 met the inclusion criteria and completed the assessment, with a participation rate of 92.54%. The demographic characteristics are presented in Table 2. The mean age of the study sample was 31.4 (standard deviation (SD) = 5.76) years, and the majority were male, accounting for 97.9% of the sample. In addition, 41.9% of the participants had a college degree or above, and the mean working duration was 8.13 (SD = 6.02) years. The mean PHQ-9 and GAD-7 total scores were 4.35 (SD = 5.86) and 2.89 (SD = 4.67), respectively, while the score of global quality of life was 6.64 (SD = 1.71).

### Prevalence and correlates of internet addition

The prevalence of IA (IAT-20 total score of ≥ 50) was 13.1% (n = 454; 95% CI: 11.9–14.2%). Details of the IAT-20 item scores are shown in Table 1. As shown in Table 2, IFSO with IA were positively associated with having a bachelor or higher education (P = 0.044), married status (P = 0.047), an annual income of less than 100,000 RMB (P = 0.004) and longer work duration (years) (P = 0.008) when compared to those without IA. Additionally, compared with IFSO without IA, those with IA had higher PHQ-9 (P < 0.001) and GAD-7 (P < 0.001) total scores, but had lower global QOL (P < 0.001). After controlling for variables with significant inter-group differences in univariate analyses, the QOL in IFSO with IA (F_(1, 3,475)_ = 15.298, P < 0.001) remained lower than those without IA. A binary logistic regression analysis revealed independent associations between more severe depressive symptoms (OR = 1.105; P < 0.001) and anxiety symptoms (OR = 1.102; P < 0.001) with a higher risk of IA. In addition, participants with an annual income of more than 100,000RMB (OR = 0.728; P = 0.027) exhibited a lower risk of IA (Table 3).

**Table 1:** Item statistics of the internet addiction test

	Items	Abbreviations	Mean (SD)	EI^a^	Predictability
1	Stay online longer than you intend	IAT1	2.09 (1.22)	0.83	0.56
2	Neglect chores to spend more time online	IAT2	1.77 (1.05)	1.07	0.68
3	Prefer the excitement online to the time with others	IAT3	1.76 (1.08)	0.81	0.56
4	Form new relationships with online users	IAT4	1.55 (0.93)	0.59	0.51
5	Others complain about your time spent online	IAT5	1.67 (0.99)	0.93	0.64
6	School grades suffer due to internet use	IAT6	1.41 (0.84)	0.99	0.79
7	Check email/SNS before doing things you need to do	IAT7	1.50 (0.91)	0.81	0.64
8	Job performance or productivity suffer because of the Internet	IAT8	1.44 (0.86)	1.12	0.78
9	Become defensive/secretive about the internet use	IAT9	1.77 (1.10)	0.72	0.51
10	Soothe disturbing thoughts using the Internet	IAT10	1.65 (1.01)	0.94	0.66
11	Anticipation for future online activities	IAT11	1.55 (0.95)	1.06	0.73
12	Life boring and empty without the Internet	IAT12	1.63 (1.01)	0.92	0.64
13	Snap or act annoyed if bothered while being online	IAT13	1.48 (0.88)	1.01	0.78
14	Sleep loss due to late-night logins	IAT14	1.63 (1.00)	0.92	0.69
15	Preoccupation with the Internet	IAT15	1.54 (0.91)	1.11	0.78
16	Request an extension for longer time spent online	IAT16	1.59 (0.96)	1.04	0.75
17	Failure to cut down the time spent online	IAT17	1.50 (0.91)	1.05	0.78
18	Conceal the amount of time spent online	IAT18	1.44 (0.87)	1.04	0.80
19	Spend more time online over going out with others	IAT19	1.48 (0.91)	0.97	0.74
20	Depressed/moody/nervous only while being offline	IAT20	1.42 (0.86)	1.09	0.82

*EI* expected influence, *IAT* Internet Addiction Test, *SD* standard deviation

^a^The value of EI is shown as raw data

**Table 2:** Characteristics of Inflight security officers with and without Internet addiction

Variables	Total (N = 3,475)		Without Internet Addiction (N = 3,021)		With Internet Addiction (N = 454)		Univariable analysis		
	N	%	N	%	N	%	$\chi^{2}$	*df*	*p*
Male	3,402	97.9	2,956	97.8	446	98.2	0.291	1	0.589
Bachelor or above education	1,456	41.9	1,246	41.2	210	46.3	4.071	1	**0.044**
Married status	2,189	63.0	1,884	62.4	305	67.2	3.929	1	**0.047**
Yearly income ≤ RMB^b^ 100,000	2,620	75.4	2,253	74.6	367	80.8	8.335	1	**0.004**
	Mean	*SD*	Mean	*SD*	Mean	*SD*	*Z*	*df*	*p*
Age (years)	31.40	5.76	31.34	5.71	31.79	6.05	− 1.550	–*	0.121
Work duration (years)	8.13	6.02	8.03	5.93	8.83	6.59	− 2.662	–*	**0.008**
PHQ9-total	4.35	5.86	3.38	4.82	10.78	7.79	− 27.688	–*	** < 0.001**
GAD7-total	2.89	4.67	2.12	3.78	8.05	6.42	− 27.913	–*	** < 0.001**
Global quality of life	6.64	1.71	6.81	1.63	5.47	1.80	16.082	–*	** < 0.001**

Bolded values: < 0.05

*df* degree of freedom, *GAD-7* the 7-item Generalized Anxiety Disorder, *IAT-20* the 20-item Internet Addiction Test, *PHQ-9* the 9-item Patient Health Questionnaire, *SD* standard deviation

^*^Mann–Whitney U test

^b^1 RMB = 0.155 USD

**Table 3:** Binary logistic regression analysis of Internet addiction in Inflight security officers (N = 3,475)

Variables	Binary logistic regression analysis		
	*p*	*OR*	95% *CI*
Bachelor or above education	0.923	0.989	0.788–1.241
Married status	0.060	0.792	0.621–1.009
Yearly income ≤ RMB^b^ 100,000	**0.027**	0.728	0.549–0.965
Work duration (years)	0.366	0.991	0.972–1.010
PHQ9-total	** < 0.001**	1.105	1.068–1.144
GAD7-total	** < 0.001**	1.102	1.058–1.149

Bolded values: < 0.05

*CI* confidence interval, *OR* odds ratio

^b^1 RMB = 0.155 USD

### Network structure and centrality

**Fig. 1:**
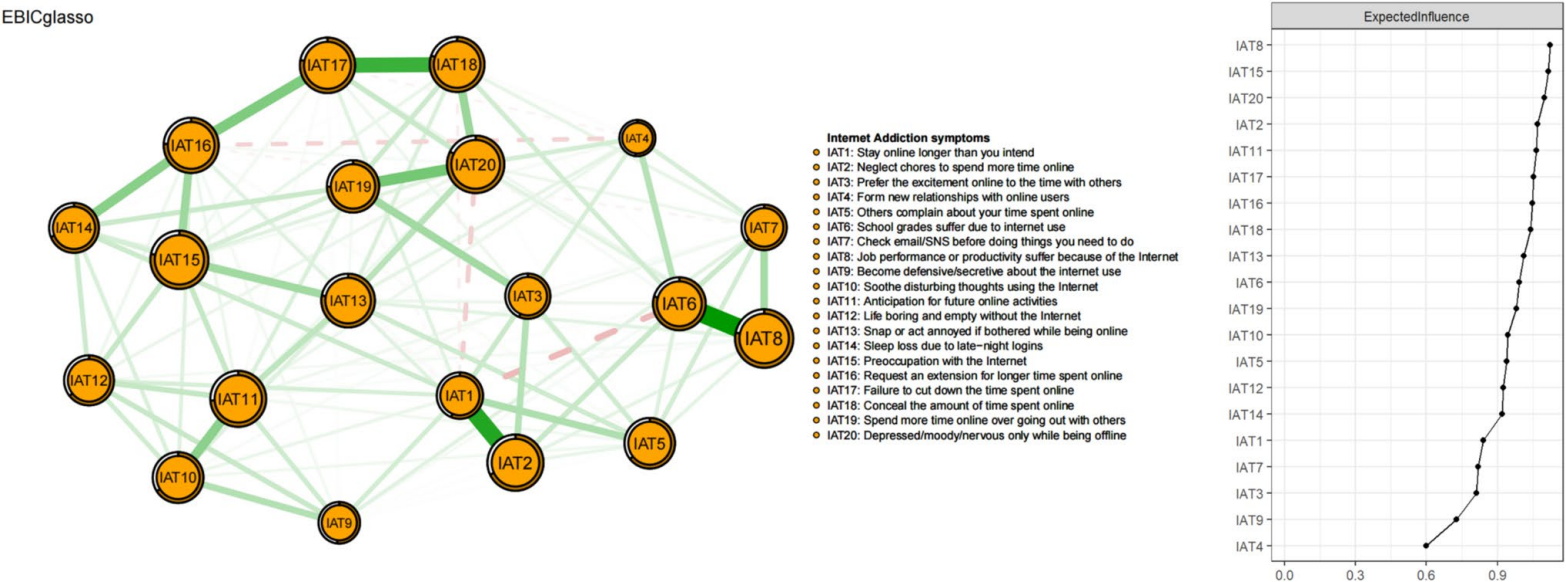
Network structure of internet addiction symptoms in inflight security officers

Figure 1 displays the network structure of IA symptoms. Of the 190 edges estimated, 128 were non-zero edges, with the mean weight of 0.05. “School grades suffer due to internet use” (IAT6)—“Job performance or productivity suffer because of the Internet” (IAT8) were the strongest edge (weight:0.60), followed by “Stay online longer than you intend” (IAT1)—“Neglect chores to spend more time online” (IAT2) (weight: 0.52), and “Failure to cut down the time spent online” (IAT17)—“Conceal the amount of time spent online” (IAT18) (weight: 0.46).

The EI values in the IAT network are plotted in the right panel of Fig. 1. Table 1 shows the raw data of EI values. “Job performance or productivity suffer because of the Internet” (IAT8) was the most central symptom with the highest EI value (EI: 1.12), followed by “Preoccupation with the Internet” (IAT15) (EI:1.11) and “Depressed/moody/nervous only while being offline” (IAT20) (EI: 1.09). In addition, the average predictability values of nodes in the network model was 0.69 (SD = 0.09), indicating that on average 69% of the variance of each node could be potentially explained by neighboring nodes.

**Fig. 2:**
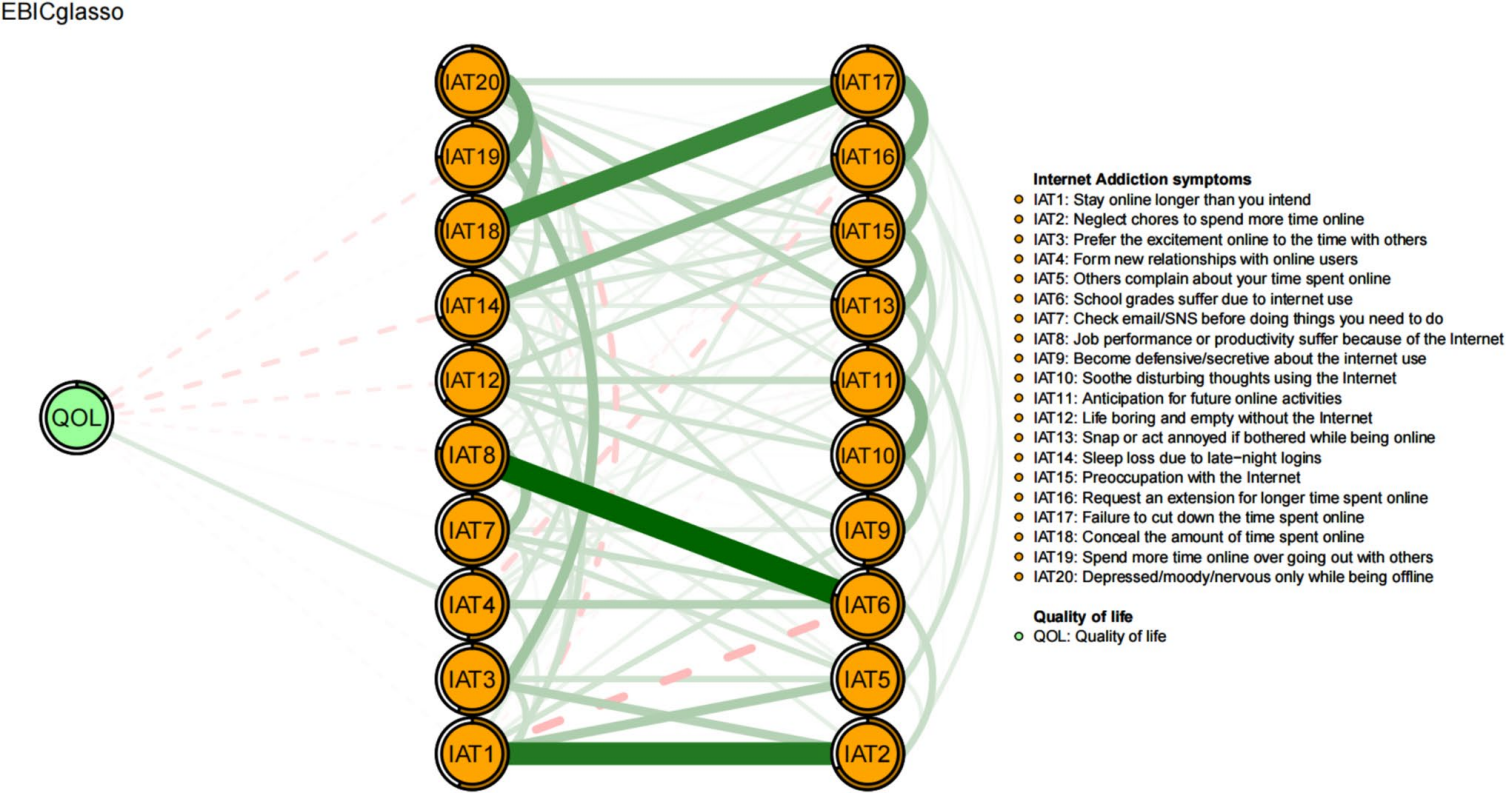
Flow network of quality of life and Internet addiction symptoms. Note: items in the middle of the figure indicate the direct connection to quality of life and items located on the right represent the indirect connection

Figure 2 shows the flow network of connections between QOL and the symptoms of IA. Among the IA symptoms directly related to QOL, the strongest negative edges were observed in “Sleep loss due to late-night logins” (IAT14) (average edge weight = −0.08), followed by “Spend more time online over going out with others” (IAT19) (average edge weight = −0.06). Additionally, “Form new relationships with online users” (IAT4) exhibited the strongest positive correlation with QOL (average edge weight = 0.08).

### Network stability and accuracy

The stability of the network model is shown in Figure S1 based on the case-dropping bootstrap method. The CS-coefficients value for EI was 0.75, indicating that the network could still maintain stability after 75% of the samples were dropped. Moreover, Figure S2 shows the accuracy of the network model. The value range of the estimation of edge weights by Bootstrap 95% CIs were narrow, indicating that the primary results were reliable.

As shown in Figure S3, the bootstrapped difference test results indicated that the most central symptoms in the network model were significantly different from most of other nodes, indicating that the EI results were robust. In addition, the bootstrapped difference test results of edge weights showed that the difference between the strongest edges and most edges were statistically significant, which supported the accuracy and reliability of the network model (Figure S4).

## Discussion

This was the first study to examine the prevalence of IA as well as its associated factors, relationship with QOL and network structure among IFSO working in commercial airlines in China. Due the specific requirements needed for recruiting IFSO, most of the participants were male (97.9%) in this study. We found that IA was common among IFSO, particularly in those who had more severe anxiety and depression symptoms, and poor economic status. Network analysis identified that “Job performance or productivity suffer because of the Internet” (IAT8), “Preoccupation with the Internet” (IAT15) and “Depressed/moody/nervous only while being offline” (IAT20)"were the most central nodes. In addition, in IA-QOL network models of IFSO, “Sleep loss due to late-night logins” (IAT14) and “Spend more time online over going out with others” (IAT19) had the most negative correlation, while “Form new relationships with online users” (IAT4) had the strongest positive correlation with QOL.

We found that the prevalence of IA (IAT-20 ≥ 50) was 13.1% (95% CI: 11.9–14.2%) among IFSO, which is significantly higher than the prevalence reported in a previous systematic review [7], which ranged from 1.2% (Internet users in the UK) [73] to 9.7% (college students in Turkey) [74], as well as from 8.0% among airline pilots (95% CI: 7.3–8.6%) [75] to 11.3% among the general population (95% CI: 10.1%–12.5%) [76]. Although IA was measured using the IAT in all the studies, the reasons for the differences in prevalence might be due to the differences sample selection as well as Internet usage behaviors between various geographic locations [47, 77, 78]. However, the IA prevalence in our study was lower than the prevalence among adolescents (20.3%) and children (13.8%) in Korea [39], which might be related to several factors in children and adolescents such as the lack of self-control, perceived parental monitoring stress [42], reduced emotional stability [79], and low adaptability and conscientiousness [41].

Past studies [78, 80] found that IA was positively related to both depressive and anxiety symptoms, which was supported by our study findings. A bidirectional relationship between IA and other psychiatric disorders (e.g., depression and anxiety) has been previously demonstrated [6, 81, 82]. On the one hand, those with mental health problems might have less willingness to seek help in-person due to stigma or discrimination [83], and might be more willing to seek online help using e-mental health resources [84], thereby increasing their Internet use and the risk of IA. On the other hand, IA might also directly increase the risk of depression and anxiety [85]. For example, the survey in Korean adults found that IA group had a higher risk of depression and anxiety compared to the control group [86]. The results of this study showed that participants with lower-income levels have a higher risk of IA, which is consistent with previous findings that low-income was a predictor of IA [24, 87]. Financial difficulties have been reported as a risk factor associated with IA in adults [88]. Compared to other social activities, Internet use is a cheaper and more convenient activity for low-income IFSO with limited social support [89]. Hence, although increased Internet use could compensate for their perceived stress and isolation, turning to the Internet for social support might also increase the risk of IA [89].

“Job performance or productivity suffer because of the Internet” (IAT8) was the most influential node of the IA network, which as similar to the network model of IA among younger (aged 14–24 years) [90] and MDD patients [38]. Our results are also consistent with past research that Internet overuse might negatively influence daily work performance and reduce employee productivity [91]. Due to the work requirements of IFSO, IA may affect their work ability by interfering with their sleep patterns [92]. Frequent time zone changes, long-distance flights and changing shift work for IFSO will likely disrupt their work and rest schedules, which can result in higher risk of sleep problems than the general population [93]. Hence, excessive Internet use coupled with chronic irregular work and rest patterns may aggravate their sleep problems and negatively affect their work performance [94]. However, our findings might be unique to the occupational characteristics IFSO, which might limit the generalizability of our findings to other populations such as unemployed individuals or other non-working populations.

“Preoccupation with the Internet” (IAT15), defined as “a preoccupation with the Internet while offline or fantasizing about being online” [28], was also the key influential node in the network model. The finding is similar to past researches reported among adolescents (aged 12–20 years), young adults (aged 14–24 years) [90], pregnant and postpartum women [65] and depressed patients [95]. “Preoccupation with the Internet” (IAT15) has also been found in Previous research [40] to be a key node in activating other IA symptoms. IA patients usually start with excessive attention to the Internet and when they are offline, they often miss the use of the Internet, being unable to control their own behavior, all of which can influence their daily work and life [96, 97]. In the virtual environment of the Internet, people do not need to be seen, they can freely define their identity and self-image in any way [98]. Moreover, as IFSO often work in a closed, high-pressure, and constantly changing environment, such conditions may limit their opportunities for social interaction, increase their feelings of loneliness, and lead to a decline in social skills [3, 5, 6]. Additionally, the frequently changing work environment and time zones contribute to a sense of detachment from the real world, making them more inclined to seek social fulfillment within virtual online spaces [75, 99].

“Depressed/moody/nervous only while being offline” (IAT20) was another central symptom, which is consistent with the findings of AI network models among nursing students [34] and adolescents with autism spectrum disorder [37]. This finding may be attributed to possible withdrawal symptoms of IA while being offline which may include depressive and anxiety symptoms [100]. Further, as IFSO often use the Internet to cope with their depressive and anxious emotions resulting from their work pressure, there may be an increased tendency for anxiety and depressive symptoms when they are offline [101, 102].

Similar to our study findings, as previously reported, individuals with IA tend to experience more physical pain, cognitive impairment and psychological problems [23], poor sleep quality, financial difficulties [88], poor interpersonal relationships [81, 103], and decreased social adjustment [104], all of which may lower QOL.

In addition, our study found that “Sleep loss due to late-night logins” (IAT14) and “Spend more time online over going out with others” (IAT19) were directly and most negatively associated with QOL, which aligns with past studies showing that poorer QOL was significantly related to various IA domains [38, 65]. IA may lead to sleep problems and neglect of real-life social interactions, thereby reducing QOL. Although online gaming can create short term positive emotions for IA patients, they are often followed by a strong sense of emptiness and loneliness [87]. Previous studies have found that staying up late to use mobile devices, such as being addicted to online games, could lead to poor sleep quality, insomnia, sleep disorders and other sleep problems [105, 106], all of which could have a negative impact on daily work and QOL. Furthermore, when individuals are addicted to using the Internet to participate in social media or play online games, they are likely to socially disconnect from their friends and family in the real world and have impairment in their activities of daily life and work [91, 107, 108], which may in turn lower their QOL.

In the flow network model, “form new relationships with online users” (IAT4) showed the strongest positive correlation with QOL, which is consistent with the IA-QOL network model among patients with MDD [38]. In the immediate aftermath of the COVID-19 pandemic, during which airlines in China had maintained strict public health measures, including social distancing and conducted this study. These measures led to reduced offline social activities but increased online social interactions to enhance social contacts [109, 110], which might improve QOL, particularly in social domains.

This study has some strengths. First, we have a large sample size and high participation rate. Second, we have nationally representative sample of the IFSO across China. Third, we used the network analysis to explore the central node of IA. However, several limitations should be noted. First, due to the cross-sectional study design, causality between IA symptoms and other variables, such as depression, anxiety and QOL, could not be examined. Future longitudinal studies on the causal relationship between IA and anxiety, depression and QOL are needed to clarify whether IA leads to poor mental health or vice versa. Second, since the IAT-20 questionnaire was a self-report assessment, there might be recall bias. Third, certain relevant variables, such as substance use disorders (e.g., tobacco and alcohol use), primary online activities and behavior patterns, should be included in further research. Finally, convenience samples and the high proportion of male participants (97.9%) in this study limit the representativeness of the study sample.

In conclusion, our network study found that IA was common among IFSO, particularly among those with lower annual income and depressive and anxiety symptoms. To address IA in clinical practice, certain psychosocial interventions, such as CBT, have been widely used effectively [111]. “Job performance or productivity suffer because of the Internet” (IAT8), “Preoccupation with the Internet” (IAT15) and “Depressed/moody/nervous only while being offline” (IAT20) were identified as the most central IA symptoms. To reduce the negative outcomes associated with IA in IFSO and to improve their QOL, such symptoms should be prioritized in both treatment and preventive strategies.

## Supplementary Information

Below is the link to the electronic supplementary material.

Supplementary file1 (DOCX 1024 KB)

## Data Availability

According to local ethical regulations, the data involved in this study are not publicly available.

## References

[1] Jinyu Pan (2024) The National Civil Aviation Safety Work Conference was held in Beijing (in Chinese). Available from: http://www.caacnews.com.cn/1/1/202401/t20240105_1373726.html, China Civil Aviation News, Beijing. Accessed 22 Feb 2025

[2] Min Xiao (2024) The National Civil Aviation Work Conference was held in Beijing (in Chinese). Available from: http://www.caacnews.com.cn/tt/202401/t20240104_1373688.html. China Civil Aviation News Accessed 22 Feb 2025

[3] Abeyratne R (2014) The conundrum of the inflight security officer (IFSO). J Transp Secur 7(3):199–207. 10.1007/s12198-014-0139-1

[4] Huang J, Mian Lu (2018) Discussion on the important role of physical quality of civil aviation air safety personnel to aviation safety (in Chinese). Sport Goods Technol 17:154–155

[5] Tie F (2016) Analysis of air safety personnel physical quality reserve and improvement (in Chinese). New Silk Road (late) 07:132. 10.19312/j.cnki.61-1499/c.2016.07.089

[6] Carli V, Durkee T, Wasserman D, Hadlaczky G, Despalins R, Kramarz E, Wasserman C, Sarchiapone M, Hoven CW, Brunner R, Kaess M (2013) The association between pathological internet use and comorbid psychopathology: a systematic review. Psychopathology 46(1):1–13. 10.1159/000337971 22854219 10.1159/000337971

[7] Kuss D, Griffiths M, Karila L, Billieux J (2014) Internet addiction: a systematic review of epidemiological research for the last decade. Curr Pharma Des 20(25):4026–4052 10.2174/13816128113199990617 24001297

[8] Anderson EL, Steen E, Stavropoulos V (2017) Internet use and problematic internet use: a systematic review of longitudinal research trends in adolescence and emergent adulthood. Int J Adolesc Youth 22(4):430–454. 10.1080/02673843.2016.1227716

[9] Christakis DA (2010) Internet addiction: a 21st century epidemic? BMC Med 8:3. 10.1186/1741-7015-8-61 20955578 10.1186/1741-7015-8-61 PMC2972229

[10] Charlton JP (2002) A factor-analytic investigation of computer “addiction” and engagement. Br J Psychol 93:329–344. 10.1348/000712602760146242 12230834 10.1348/000712602760146242

[11] Shaw M, Black DW (2008) Internet addiction: Definition, assessment, epidemiology and clinical management. CNS Drugs 22(5):353–365. 10.2165/00023210-200822050-00001 18399706 10.2165/00023210-200822050-00001

[12] American Psychiatric Association (2013) Diagnostic and statistical manual of mental disorders, 5th edn. American Psychiatric Association, Arlington, VA

[13] Kaess M, Klar J, Kindler J, Parzer P, Brunner R, Carli V, Sarchiapone M, Hoven CW, Apter A, Balazs J (2021) Excessive and pathological Internet use–Risk-behavior or psychopathology? Addict Behav 123:107045 34332272 10.1016/j.addbeh.2021.107045

[14] Jamieson K, Romer D (2005) Treating and preventing adolescent mental health disorders: What we know and what we don’t know. Oxford University Press, New York, NY. 10.1093/med-psych/9780199928163.001.0001

[15] Pan YC, Chiu YC, Lin YH (2020) Systematic review and meta-analysis of epidemiology of internet addiction. Neurosci Biobehav Rev 118:612–622. 10.1016/j.neubiorev.2020.08.013 32853626 10.1016/j.neubiorev.2020.08.013

[16] Liu X, Gui Z, Chen Z M, Feng Y, Wu X D, Su Z, Cheung T, Ungvari GS, Liu X C, Yan Y R (2025) Global prevalence of internet addiction among university students: a systematic review and meta-analysis. Curr Opin Psychiatr, 38(3): 182-199. DOI: 10.1097/YCO.0000000000000994 10.1097/YCO.0000000000000994 40009750

[17] Ayas T, Horzum M (2013) Relation between depression, loneliness, self-esteem and internet addiction. Education 133(3):283–290

[18] Buneviciene I, Bunevicius A (2021) Prevalence of internet addiction in healthcare professionals: systematic review and meta-analysis. Int J Soc Psychiatr 67(5):483–491. 10.1177/0020764020959093 10.1177/0020764020959093 32962501

[19] Chang FC, Chiu CH, Miao NF, Chen PH, Lee CM, Chiang JT, Pan YC (2015) The relationship between parental mediation and Internet addiction among adolescents, and the association with cyberbullying and depression. Compr Psychiat 57:21–28. 10.1016/j.comppsych.2014.11.013 25487108 10.1016/j.comppsych.2014.11.013

[20] Díaz Cárdenas S, Arrieta Vergara K, Simancas-Pallares M (2019) Internet addiction and academic performance in dental students. Revista Colombiana de Psiquiatría (English ed) 48(4):198–207. 10.1016/j.rcpeng.2018.03.009 10.1016/j.rcp.2018.03.002 31779870

[21] Li X, Luo XM, Zheng RM, Jin X, Mei LL, Xie XY, Gu HT, Hou F, Liu LF, Luo X, Meng H, Zhang JJ, Song RR (2019) The role of depressive symptoms, anxiety symptoms, and school functioning in the association between peer victimization and internet addiction: a moderated mediation model. J Affect Disord 256:125–131. 10.1016/j.jad.2019.05.080 31176184 10.1016/j.jad.2019.05.080

[22] Brand M, Young KS, Laier C (2014) Prefrontal control and Internet addiction: a theoretical model and review of neuropsychological and neuroimaging findings. Front Hum Neurosci 8:375 24904393 10.3389/fnhum.2014.00375 PMC4034340

[23] Kurniasanti KS, Assandi P, Ismail RI, Nasrun MWS, Wiguna T (2019) Internet addiction: a new addiction? Med J Indonesia 28(1):82–91

[24] Wu CST, Wong HT, Yu KF, Fok KW, Yeung SM, Lam CH, Liu KM (2016) Parenting approaches, family functionality, and internet addiction among Hong Kong adolescents. BMC Pediatr 16:10. 10.1186/s12887-016-0666-y 27538688 10.1186/s12887-016-0666-y PMC4991114

[25] Du Y-s, Jiang W, Vance A (2010) Longer term effect of randomized, controlled group cognitive behavioural therapy for Internet addiction in adolescent students in Shanghai. Aust N Z J Psychiatry 44(2):129–134 20113301 10.3109/00048670903282725

[26] Camardese G, Leone B, Walstra C, Janiri L, Guglielmo R (2017) Pharmacological Treatment of Internet Addiction. In: Montag C, Reuter M (eds) Internet Addiction: Neuroscientific Approaches and Therapeutical Implications Including Smartphone Addiction, 2nd Edition. Studies in Neuroscience Psychology and Behavioral Economics. Springer International Publishing Ag, Cham, pp 231–245. 10.1007/978-3-319-46276-9_14

[27] Jiang Q, Chen Z, Zhang Z, Zuo C (2023) Investigating links between Internet literacy, Internet use, and Internet addiction among Chinese youth and adolescents in the digital age. Front Psych 14:1233303 10.3389/fpsyt.2023.1233303 PMC10513100 37743978

[28] Young KS (1998) Internet addiction: the emergence of a new clinical disorder. Cyberpsychol Behav 1(3):237–244. 10.1089/cpb.1998.1.237

[29] Borsboom D (2017) A network theory of mental disorders. World Psychiatry 16(1):5–13. 10.1002/wps.20375 28127906 10.1002/wps.20375 PMC5269502

[30] Borsboom D, Cramer AOJ (2013) Network analysis: an integrative approach to the structure of psychopathology. Ann Rev Clin Psychol 9:91–121 23537483 10.1146/annurev-clinpsy-050212-185608

[31] Murri MB, Amore M, Respino M, Alexopoulos GS (2020) The symptom network structure of depressive symptoms in late-life: results from a European population study. Mol Psychiatr 25(7):1447–1456. 10.1038/s41380-018-0232-0 10.1038/s41380-018-0232-0 30171210

[32] Epskamp S, Kruis J, Marsman M (2017) Estimating psychopathological networks: be careful what you wish for. PLoS ONE 12(6):13. 10.1371/journal.pone.0179891 10.1371/journal.pone.0179891 PMC5482475 28644856

[33] Cai H, Zhao YJ, He F, Li SY, Li ZL, Zhang WY, Zhang Y, Cheung T, Ng CH, Sha S, Xiang YT (2023) Internet addiction and residual depressive symptoms among clinically stable adolescents with major psychiatric disorders during the COVID-19 pandemic: a network analysis perspective. Transl Psychiatry 13(1):9. 10.1038/s41398-023-02468-5 37270593 10.1038/s41398-023-02468-5 PMC10238780

[34] Cai H, Xi HT, An FR, Wang ZW, Han L, Liu S, Zhu QQ, Bai W, Zhao YJ, Chen L, Ge ZM, Ji MM, Zhang HY, Yang BX, Chen P, Cheung T, Jackson T, Tang YL, Xiang YT (2021) The association between internet addiction and anxiety in nursing students: a network analysis. Front Psych 12:10. 10.3389/fpsyt.2021.723355 10.3389/fpsyt.2021.723355 PMC8424202 34512421

[35] Yang Y, Zhang EL, Liu Y, Ge X, Su Z, Cheung T, Ng CH, Xiang M, Xiang Y-T (2023) Network analysis of suicidality and internet addiction symptoms among Chinese primary and secondary school students. J Affect Disord 339:145–152 37437741 10.1016/j.jad.2023.07.030

[36] Huang S, Lai X, Xue Y, Zhang C, Wang Y (2021) A network analysis of problematic smartphone use symptoms in a student sample. J Behav Addict 9(4):1032–1043 10.1556/2006.2020.00098 PMC8969737 33372911

[37] Hirota T, McElroy E, So RH (2021) Network analysis of internet addiction symptoms among a clinical sample of Japanese adolescents with autism spectrum disorder. J Autism Dev Disord 51(8):2764–2772. 10.1007/s10803-020-04714-x 33040268 10.1007/s10803-020-04714-x

[38] Bai W, Cai H, Wu SQ, Zhang L, Feng KX, Li YC, Liu HZ, Du XD, Zeng ZT, Lu CM, Mi WF, Zhang L, Ding YH, Yang JJ, Jackson T, Cheung T, An FR, Xiang YT (2022) Internet addiction and its association with quality of life in patients with major depressive disorder: a network perspective. Transl Psychiatry 12(1):7. 10.1038/s41398-022-01893-2 35379778 10.1038/s41398-022-01893-2 PMC8977829

[39] Ha JH, Yoo HJ, Cho IH, Chin B, Shin D, Kim JH (2006) Psychiatric comorbidity assessed in Korean children and adolescents who screen positive for Internet addiction. J Clin Psychiatry 67(5):821–826. 10.4088/JCP.v67n0517 16841632 10.4088/jcp.v67n0517

[40] Cai H, Bai W, Sha S, Zhang L, Chow IHI, Lei SM, Lok GKI, Cheung T, Su ZH, Hall BJ, Smith RD, Xiang YT (2022) Identification of central symptoms in Internet addictions and depression among adolescents in Macau: a network analysis. J Affect Disord 302:415–423. 10.1016/j.jad.2022.01.068 35065088 10.1016/j.jad.2022.01.068

[41] Kuss DJ, van Rooij AJ, Shorter GW, Griffiths MD, van de Mheen D (2013) Internet addiction in adolescents: Prevalence and risk factors. Comput Hum Behav 29(5):1987–1996. 10.1016/j.chb.2013.04.002

[42] Yen CF, Ko CH, Yen JY, Chang YP, Cheng CP (2009) Multi-dimensional discriminative factors for Internet addiction among adolescents regarding gender and age. Psychiatry Clin Neurosci 63(3):357–364. 10.1111/j.1440-1819.2009.01969.x 19566768 10.1111/j.1440-1819.2009.01969.x

[43] Tran BX, Huong LT, Hinh ND, Nguyen LH, Le BN, Nong VM, Thuc VTM, Tho TD, Latkin C, Zhang MWB, Ho RCM (2017) A study on the influence of internet addiction and online interpersonal influences on health-related quality of life in young Vietnamese. BMC Public Health 17:8. 10.1186/s12889-016-3983-z 28143462 10.1186/s12889-016-3983-z PMC5282902

[44] Zeng JJ, Lai JH, Liu XF (2022) How servant leadership motivates young university teachers’ workplace well-being: the role of occupational commitment and risk perception. Front Psychol 13:11. 10.3389/fpsyg.2022.996497 10.3389/fpsyg.2022.996497 PMC9583247 36275314

[45] Lu TC, Wang CX, Chen HW, Tao BL, Jiang YY, Sui HR, Yan J (2022) Relationship between university students’ physical activity and mobile phone dependence: Mediating effect of subjective well-being and moderating effect of psychological capital. Front Psychol 13:10. 10.3389/fpsyg.2022.983487 10.3389/fpsyg.2022.983487 PMC9835677 36643699

[46] Karatoprak S, Donmez YE (2020) Internet addiction and comorbid pyschiatric disorders in adolescents. Ann Med Res 27:504

[47] Chang MK, Law SPM (2008) Factor structure for young’s internet addiction test: a confirmatory study. Comput Hum Behav 24(6):2597–2619. 10.1016/j.chb.2008.03.001

[48] Yang JJ, Bai W, Guo T, Zhang L, Li YC, Liu HZ, Du XD, Cai H, Balbuena L, An FR, Xiang YT (2022) The prevalence of internet addiction and its association with quality of life among clinically stable patients with major depressive disorder. J Affect Disord 314:112–116. 10.1016/j.jad.2022.06.067 35777497 10.1016/j.jad.2022.06.067

[49] Dufour M, Brunelle N, Tremblay J, Leclerc D, Cousineau M-M, Khazaal Y, Légaré A-A, Rousseau M, Berbiche D (2016) Gender difference in internet use and internet problems among Quebec high school students. Can J Psychiatr 61(10):663–668 10.1177/0706743716640755 PMC5348090 27310231

[50] Tateno M, Teo AR, Shiraishi M, Tayama M, Kawanishi C, Kato TA (2018) Prevalence rate of Internet addiction among Japanese college students: two cross-sectional studies and reconsideration of cut-off points of young’s internet addiction test in Japan. Psychiatr Clin Neurosci 72(9):723–730 10.1111/pcn.12686 29845676

[51] Zhou JY, Liu L, Xue P, Yang XR, Tang XD (2020) Mental health response to the COVID-19 outbreak in China. Am J Psychiat 177(7):574–575. 10.1176/appi.ajp.2020.20030304 32375540 10.1176/appi.ajp.2020.20030304

[52] Chen M, Sheng L, Qu S (2015) Diagnostic test of screening depressive disorder in general hospital with the patient health questionnaire (in Chinese). Chinese Mental Health 29(4):241–245

[53] Kroenke K, Spitzer RL, Williams JBW (2001) The PHQ-9 - validity of a brief depression severity measure. J Gen Intern Med 16(9):606–613. 10.1046/j.1525-1497.2001.016009606.x 11556941 10.1046/j.1525-1497.2001.016009606.x PMC1495268

[54] Spitzer RL, Kroenke K, Williams JBW, Lowe B (2006) A brief measure for assessing generalized anxiety disorder - the GAD-7. Arch Intern Med 166(10):1092–1097. 10.1001/archinte.166.10.1092 16717171 10.1001/archinte.166.10.1092

[55] He XY, Li CB, Qian J, Cui HS, Wu WY (2010) Reliability and validity of a generalized anxiety scale in general hospital outpatients (in Chinese). Shanghai Arch Psychiatr 22(4):200–203

[56] Kroenke K, Spitzer RL, Williams JBW, Lowe B (2010) The patient health questionnaire somatic, anxiety, and depressive symptom scales: a systematic review. Gen Hosp Psych 32(4):345–359. 10.1016/j.genhosppsych.2010.03.006 10.1016/j.genhosppsych.2010.03.006 20633738

[57] Xia P, Li NX, Hau KT, Liu CJ, Lu YB (2012) Quality of life of Chinese urban community residents: a psychometric study of the mainland Chinese version of the WHOQOL-BREF. BMC Med Res Methodol 12:11. 10.1186/1471-2288-12-37 22452994 10.1186/1471-2288-12-37 PMC3364902

[58] Liu R, Xu X, Zou S, Li Y, Wang H, Yan X, Du X, Zhang L, Zhang Q, Li W (2022) Prevalence of suicidality and its association with quality of life in older patients with clinically stable psychiatric disorders in China during the COVID-19 pandemic. J Geriatr Psychiatr Neurol 35(2):237–244 10.1177/08919887221078557 PMC8899831 35246000

[59] Lin Y, Bai W, Liu H-H, Li Z-Z, Gao Z-Z, Han T, Ren H-H, Ng CH, Xiang Y-T (2023) Prevalence, correlates, and network analysis of depression and its association with quality of life in survivors with myocardial infarction during the COVID-19 pandemic. J Affect Disord 336:106–111 37247785 10.1016/j.jad.2023.05.086 PMC10224774

[60] Harper A, Power M, Grp W (1998) Development of the World Health Organization WHOQOL-BREF quality of life assessment. Psychol Med 28(3):551–558. 10.1017/s0033291798006667 9626712 10.1017/s0033291798006667

[61] R Development Core Team (2022) R: A language and environment for statistical computing. R Foundation for Statistical Computing, Vienna, Austria. Available from: https://www.R-project.org/ (Accessed 22 Feb 2025).

[62] Epskamp S, Cramer AOJ, Waldorp LJ, Schmittmann VD, Borsboom D (2012) qgraph: network visualizations of relationships in psychometric data. J Stat Softw 48(4):1–18. 10.18637/jss.v048.i04

[63] Jones P, Jones MP (2018) Package ‘networktools’. CRAN-R,

[64] Gómez-Rubio V (2017) ggplot2-elegant graphics for data analysis. J Stat Softw 77:1–3

[65] Yang Y, Zhang D-Y, Li Y-L, Zhang M, Wang P-H, Liu X-H, Ge L-N, Lin W-X, Xu Y, Zhang Y-L (2022) Prevalence, correlates, and network analysis of Internet addiction symptoms among Chinese pregnant and postpartum women. J Affect Disord 298:126–133 34715164 10.1016/j.jad.2021.10.092

[66] Abplanalp SJ, Braff DL, Light GA, Nuechterlein KH, Green MF, Gur RC, Gur RE, Stone WS, Greenwood TA, Lazzeroni LC (2022) Understanding connections and boundaries between positive symptoms, negative symptoms, and role functioning among individuals with schizophrenia: a network psychometric approach. JAMA Psychiat 79(10):1014–1022 10.1001/jamapsychiatry.2022.2386 PMC9386606 35976655

[67] Skjerdingstad N, Johnson MS, Johnson SU, Hoffart A, Ebrahimi OV (2021) Feelings of worthlessness links depressive symptoms and parental stress: a network analysis during the COVID-19 pandemic. Eur Psychiatry 64(1):e50 34311806 10.1192/j.eurpsy.2021.2223 PMC8376856

[68] Robinaugh DJ, Millner AJ, McNally RJ (2016) Identifying highly influential nodes in the complicated grief network. J Abnorm Psychol 125(6):747 27505622 10.1037/abn0000181 PMC5060093

[69] Mullarkey MC, Marchetti I, Beevers CG (2019) Using network analysis to identify central symptoms of adolescent depression. J Clin Child Adolesc Psychol 48(4):656–668 29533089 10.1080/15374416.2018.1437735 PMC6535368

[70] Haslbeck JMB, Waldorp LJ (2020) mgm: estimating time-varying mixed graphical models in high-dimensional data. J Stat Softw 93(8):46. 10.18637/jss.v093.i08

[71] Epskamp S, Fried EI (2018) Package ‘bootnet’. R package version 1

[72] Epskamp S, Borsboom D, Fried EI (2018) Estimating psychological networks and their accuracy: a tutorial paper. Behav Res Methods 50(1):195–212. 10.3758/s13428-017-0862-1 28342071 10.3758/s13428-017-0862-1 PMC5809547

[73] Morrison CM, Gore H (2010) The relationship between excessive internet use and depression: a questionnaire-based study of 1319 young people and adults. Psychopathology 43(2):121–126. 10.1159/000277001 20110764 10.1159/000277001

[74] Canan F, Ataoglu A, Ozcetin A, Icmeli C (2012) The association between Internet addiction and dissociation among Turkish college students. Compr Psychiat 53(5):422–426. 10.1016/j.comppsych.2011.08.006 22000475 10.1016/j.comppsych.2011.08.006

[75] Sun H-L, Chen P, Zhang Q, Si TL, Li Y-Z, Zhu H-Y, Zhang E, Chen M, Zhang J, Su Z, Cheung T, Ungvari GS, Jackson T, Xiang Y-T, Xiang M (2024) Prevalence and network analysis of internet addiction, depression and their associations with sleep quality among commercial airline pilots: a national survey in China. J Affect Disord. 10.1016/j.jad.2024.03.022 38484881 10.1016/j.jad.2024.03.022

[76] Li L, Xu DD, Chai JX, Wang D, Li L, Zhang L, Lu L, Ng CH, Ungvari GS, Mei SL, Xiang YT (2018) Prevalence of Internet addiction disorder in Chinese university students: a comprehensive meta-analysis of observational studies. J Behav Addict 7(3):610–623. 10.1556/2006.7.2018.53 30010411 10.1556/2006.7.2018.53 PMC6426360

[77] Ni XL, Yan H, Chen SL, Liu ZW (2009) Factors influencing internet addiction in a sample of freshmen university students in China. Cyberpsychol Behav 12(3):327–330. 10.1089/cpb.2008.0321 19445631 10.1089/cpb.2008.0321

[78] Gavurova B, Khouri S, Ivankova V, Rigelsky M, Mudarri T (2022) Internet addiction, symptoms of anxiety, depressive symptoms, stress among higher education students during the COVID-19 pandemic. Front Public Health 10:21. 10.3389/fpubh.2022.893845 10.3389/fpubh.2022.893845 PMC9237380 35774570

[79] van der Aa N, Overbeek G, Engels R, Scholte RHJ, Meerkerk GJ, Van den Eijnden R (2009) Daily and compulsive internet use and well-being in adolescence: a diathesis-stress model based on big five personality traits. J Youth Adolesc 38(6):765–776. 10.1007/s10964-008-9298-3 19636779 10.1007/s10964-008-9298-3

[80] Jaafar NS, Idris IB, Ahmad N, Hod R, Baddiri B, Hod R (2022) Internet addiction and its association with depression, anxiety, and stress symptoms among allied health students in Malaysia. Med J Indonesia 31(1):6. 10.13181/mji.oa.225820

[81] Ho RC, Zhang MWB, Tsang TY, Toh AH, Pan F, Lu YX, Cheng C, Yip PS, Lam LT, Lai CM, Watanabe H, Mak KK (2014) The association between internet addiction and psychiatric co-morbidity: a meta-analysis. BMC Psychiatry 14:10. 10.1186/1471-244x-14-183 24947851 10.1186/1471-244X-14-183 PMC4082374

[82] Ostinelli EG, Zangani C, Giordano B, Maestri D, Gambini O, D’Agostino A, Furukawa TA, Purgato M (2021) Depressive symptoms and depression in individuals with internet gaming disorder: a systematic review and meta-analysis. J Affect Disord 284:136–142. 10.1016/j.jad.2021.02.014 33592432 10.1016/j.jad.2021.02.014

[83] Brohan E, Gauci D, Sartorius N, Thornicroft G, Grp GA-ES (2011) Self-stigma, empowerment and perceived discrimination among people with bipolar disorder or depression in 13 European countries: the GAMIAN-Europe study. J Affect Disord 129(1–3):56–63. 10.1016/j.jad.2010.09.001 20888050 10.1016/j.jad.2010.09.001

[84] Semrau M, Evans-Lacko S, Koschorke M, Ashenafi L, Thornicroft G (2015) Stigma and discrimination related to mental illness in low- and middle-income countries. Epidemiol Psychiatr Sci 24(5):382–394. 10.1017/s2045796015000359 25937022 10.1017/S2045796015000359 PMC8367357

[85] Lee YS, Joo JH, Shin JY, Nam CM, Park EC (2023) Association between smartphone overdependence and generalized anxiety disorder among Korean adolescents. J Affect Disord 321:108–113. 10.1016/j.jad.2022.10.018 36283537 10.1016/j.jad.2022.10.018

[86] Kim YJ, Jang HM, Lee Y, Lee D, Kim DJ (2018) Effects of internet and smartphone addictions on depression and anxiety based on propensity score matching analysis. Int J Environ Res Public Health 15(5):10. 10.3390/ijerph15050859 10.3390/ijerph15050859 PMC5981898 29693641

[87] Leung L, Lee PSN (2012) Impact of internet literacy, internet addiction symptoms, and internet activities on academic performance. Soc Sci Comput Rev 30(4):403–418. 10.1177/0894439311435217

[88] Bakken IJ, Wenzel HG, Götestam KG, Johansson A, Oren A (2009) Internet addiction among Norwegian adults: a stratified probability sample study. Scand J Psychol 50(2):121–127. 10.1111/j.1467-9450.2008.00685.x 18826420 10.1111/j.1467-9450.2008.00685.x

[89] Lachmann B, Sariyska R, Kannen C, Stavrou M, Montag C (2017) Commuting, life-satisfaction and internet addiction. Int J Environ Res Public Health 14(10):13. 10.3390/ijerph14101176 10.3390/ijerph14101176 PMC5664677 28981452

[90] Lu JX, Zhang QH, Zhong N, Chen J, Zhai YJ, Guo L, Lu CL, Chen TZ, Jiang ZL, Zheng H (2022) Addiction symptom network of young internet users: network analysis. J Med Internet Res 24(11):13. 10.2196/38984 10.2196/38984 PMC9693725 36355402

[91] Pitichat T (2013) Smartphones in the workplace: changing organizational behavior, transforming the future. LUX J Transdiscip Writ Res Claremont Grad Univ 3(1):1–10

[92] Alimoradi Z, Lin CY, Brostrom A, Bulow PH, Bajalan Z, Griffiths MD, Ohayon MM, Pakpour AH (2019) Internet addiction and sleep problems: a systematic review and meta-analysis. Sleep Med Rev 47:51–61. 10.1016/j.smrv.2019.06.004 31336284 10.1016/j.smrv.2019.06.004

[93] Sierra JC, Delgado-Dominguez C, Carretero-Dios H (2009) Influence of sleep quality on psychopathological variables: a comparative analysis among shift workers and regular schedule workers. Rev Latinoam Psicol 41(1):121–130

[94] Pilcher JJ, Coplen MK (2000) Work/rest cycles in railroad operations: effects of shorter than 24-h shift work schedules and on-call schedules on sleep. Ergonomics 43(5):573–588. 10.1080/001401300184260 10877477 10.1080/001401300184260

[95] Cai H, Bai W, Yue Y, Zhang L, Mi WF, Li YC, Liu HZ, Du XD, Zeng ZT, Lu CM, Zhang L, Feng KX, Ding YH, Yang JJ, Jackson T, Cheung T, An FR, Xiang YT (2022) Mapping network connectivity between internet addiction and residual depressive symptoms in patients with depression. Front Psych 13:10. 10.3389/fpsyt.2022.997593 10.3389/fpsyt.2022.997593 PMC9638086 36353572

[96] Nalwa K, Anand AP (2003) Internet addiction in students: a cause of concern. Cyberpsychol Behav 6(6):653–656. 10.1089/109493103322725441 14756932 10.1089/109493103322725441

[97] Aboujaoude E (2010) Problematic internet use: an overview. World Psychiatry 9(2):85–90 20671890 10.1002/j.2051-5545.2010.tb00278.x PMC2911081

[98] Leung L (2003) Impacts of net-generation attributes, seductive properties of the Internet, and gratifications-obtained on internet use. Telematics Inform 20(2):107–129

[99] Roderick A (2023) The impact of flight on cabin crew wellness: a literature review. Senior Honors Theses. 1-41. Available from: https://digitalcommons.liberty.edu/honors/1330/(Accessed 22 Feb 2025).

[100] Young K (2009) Internet addiction: diagnosis and treatment considerations. J Contemp Psychother 39(4):241–246. 10.1007/s10879-009-9120-x

[101] Whang LSM, Lee S, Chang G (2003) Internet over-users’ psychological profiles: a behavior sampling analysis on internet addiction. Cyberpsychol Behav 6(2):143–150. 10.1089/109493103321640338 12804026 10.1089/109493103321640338

[102] Weinstein A, Dorani D, Elhadif R, Bukovza Y, Yarmulnik A, Dannon P (2015) Internet addiction is associated with social anxiety in young adults. Ann Clin Psychiatry 27(1):4–9 25696775

[103] Evcili F (2019) A study on the relationship between internet use, anxiety levels, and quality of life of Turkish pregnant women. Perspect Psychiatr Care 55(3):409–414. 10.1111/ppc.12326 30335893 10.1111/ppc.12326

[104] Cao H, Sun Y, Wan Y, Hao J, Tao F (2011) Problematic Internet use in Chinese adolescents and its relation to psychosomatic symptoms and life satisfaction. BMC Public Health 11:1–8 21995654 10.1186/1471-2458-11-802 PMC3214169

[105] Lam LT (2014) Internet gaming addiction, problematic use of the internet, and sleep problems: a systematic review. Curr Psychiatr Rep 16(4):9. 10.1007/s11920-014-0444-1 10.1007/s11920-014-0444-1 24619594

[106] Solanki SR, Shukla RP, Dave VR, Rathod VG (2023) A study on internet and gaming addiction, hikikomori trait and insomnia status among medical undergraduates at one of Cities of Western India. Indian J Community Health 35(1):66–70. 10.47203/IJCH.2023.v35i01.012

[107] Kato TA, Shinfuku N, Tateno M (2020) Internet society, internet addiction, and pathological social withdrawal: the chicken and egg dilemma for internet addiction and hikikomori. Curr Opin Psychiatr 33(3):264–270. 10.1097/yco.0000000000000601 10.1097/YCO.0000000000000601 32101902

[108] Al-Hashimi M, Razzaque A, Hamdan A, Reyad S, Badawi S, Madbouly A The impact of internet addiction on Bahraini employees’ performance. In: The Importance of New Technologies and Entrepreneurship in Business Development: In The Context of Economic Diversity in Developing Countries: The Impact of New Technologies and Entrepreneurship on Business Development, 2021. Springer, pp 142–152

[109] Ozturk FO, Ayaz-Alkaya S (2021) Internet addiction and psychosocial problems among adolescents during the COVID-19 pandemic: a cross-sectional study. Arch Psychiatr Nurs 35(6):595–601 34861951 10.1016/j.apnu.2021.08.007 PMC8424060

[110] Hu MG, Wang JF, Lin H, Ruktanonchai CW, Xu CD, Meng B, Zhang X, Carioli A, Feng YQ, Yin Q, Floyd JR, Ruktanonchai NW, Li ZJ, Yang WZ, Tatem AJ, Lai SJ (2022) Risk of severe acute respiratory syndrome coronavirus 2 (SARS-CoV-2) transmission among air passengers in China. Clin Infect Dis 75(1):E234–E240. 10.1093/cid/ciab836 34549275 10.1093/cid/ciab836 PMC9402632

[111] Young KS (2011) CBT-IA: the first treatment model for internet addiction. J Cogn Psychother 25(4):304–312. 10.1891/0889-8391.25.4.304

