# Supplementary material for: The prevalence of internet addiction and its association with quality of life among inflight security officers based on a national survey: a network analysis perspective
Source: Eur Arch Psychiatry Clin Neurosci. 2025 Jun 9;275(7):2085–97. doi: 10.1007/s00406-025-02030-y (PMC12589353; doi:10.1007/s00406-025-02030-y)
Supplement: Supplementary file 1 — Supplementary file1 (DOCX 1024 KB) [file 406_2025_2030_MOESM1_ESM.docx]

**Supplementary materials**

Supplementary Figure 1. The stability of network using the case-drop procedure (CS-coefficient=0.75)

Supplementary Figure 2. Bootstrapped confidence intervals of edge weights

Supplementary Figure 3. Estimation of node expected influence difference by bootstrapped difference test.

Supplementary Figure 4. Estimation of edge weight difference by bootstrapped difference test

**
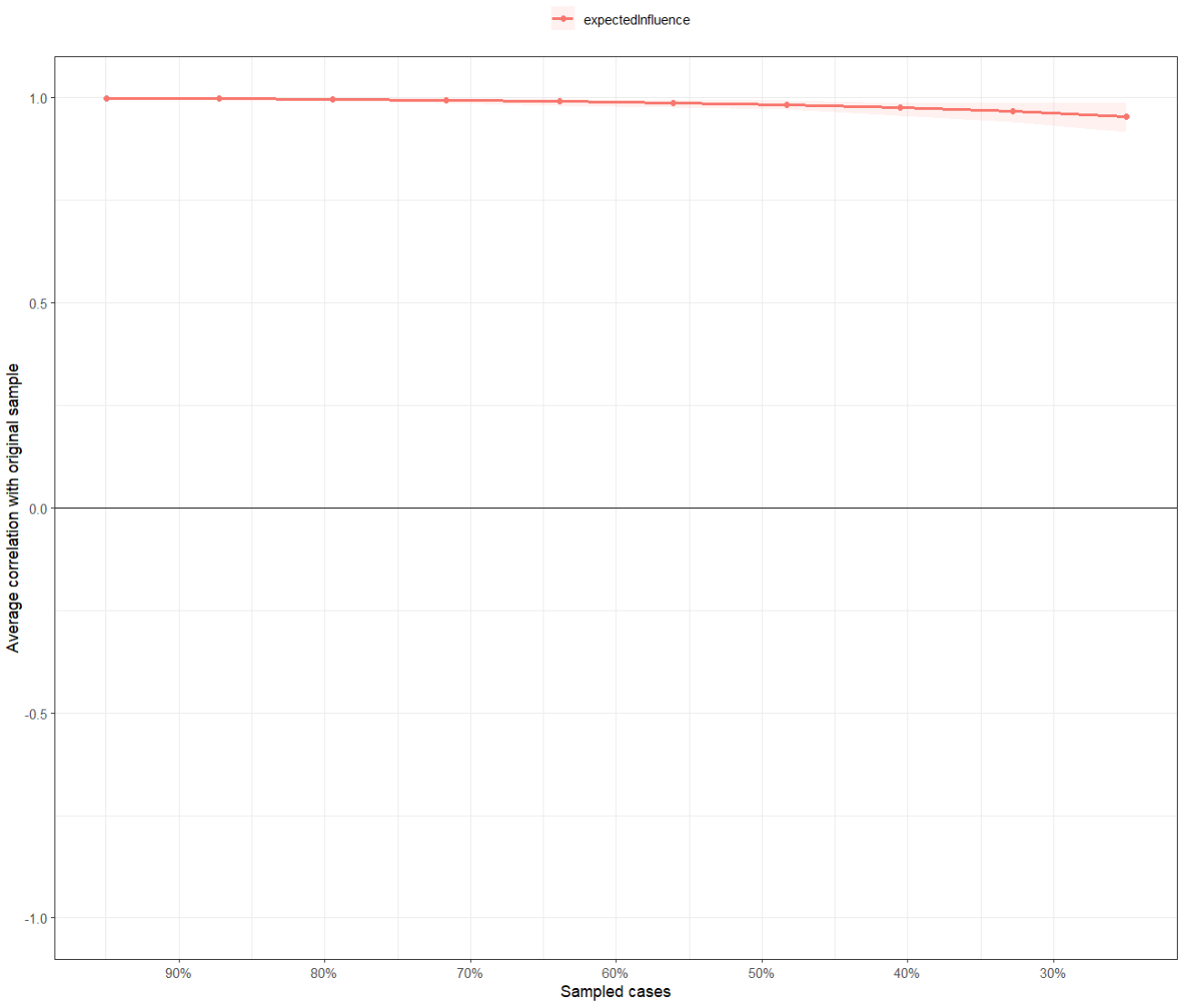
Supplementary Figure 1. The stability of network using the case-drop procedure (CS-coefficient=0.75)**


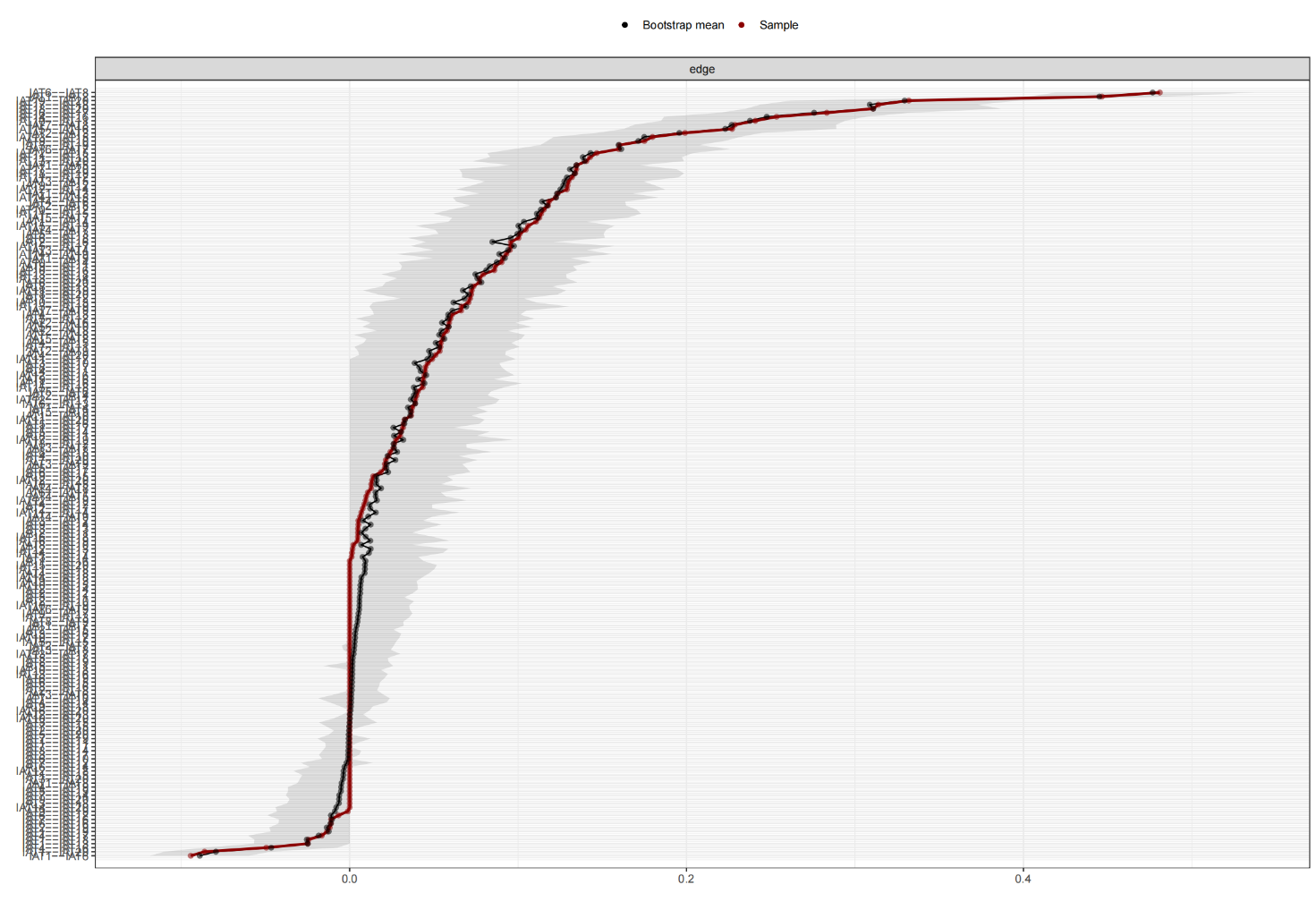


**Supplementary Figure 2. Bootstrapped confidence intervals of edge weights**

Note: The black dots indicate the values of each edge weight, ordered from the highest to the lowest value. The gray area represents the 95% Confidence Intervals of edge weights, estimated with the non-parametric bootstrap procedure (Bootnet package). Wide intervals indicate lower stability and narrow intervals indicate higher stability.


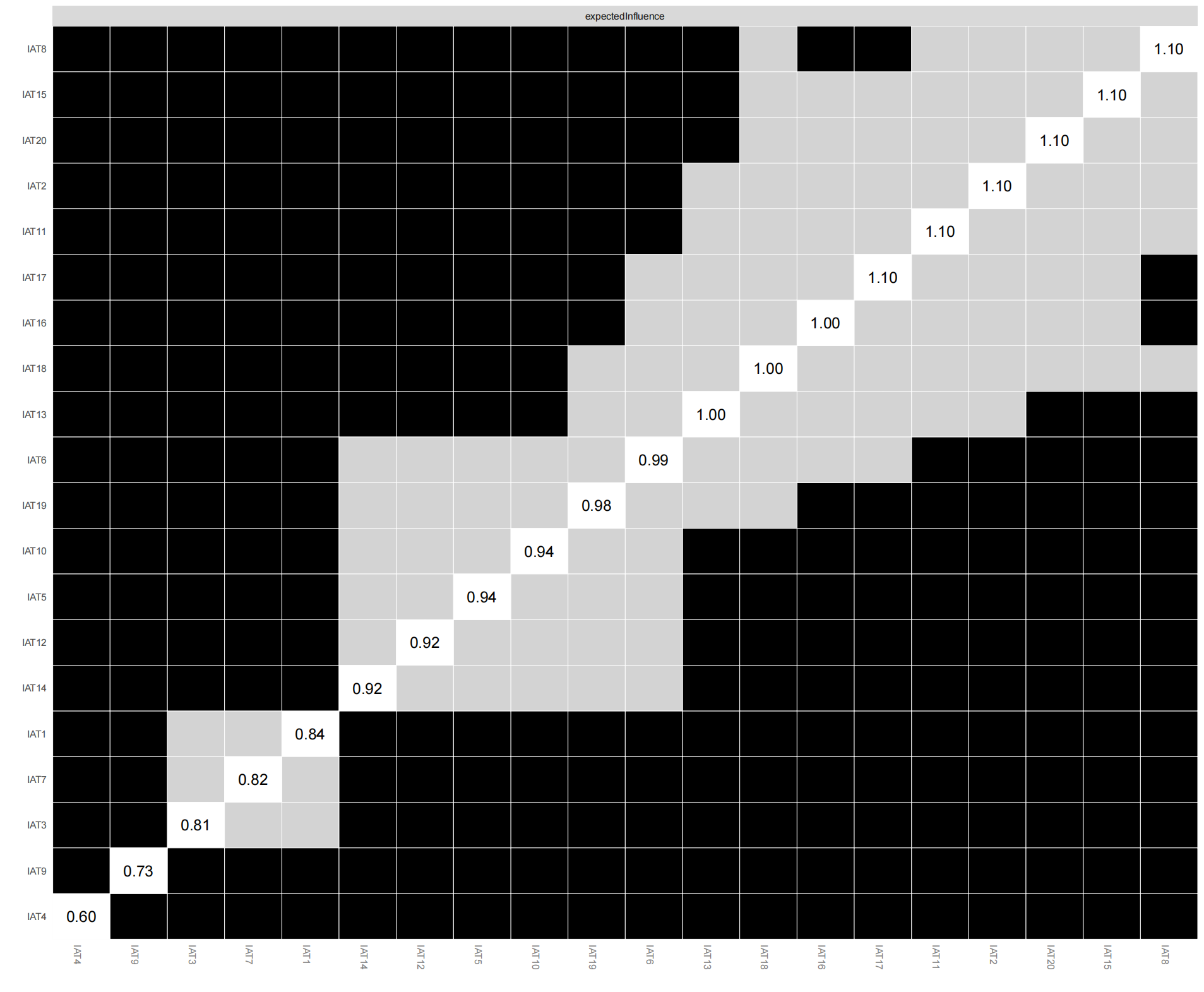


**Supplementary Figure 3. Estimation of node expected influence difference by bootstrapped difference test.**

Note: Gray boxes indicate nodes that do not significantly differ from one-another. Black boxes represent nodes that differ significantly from one another (α = 0.05). White boxes show the values of node expected influence.


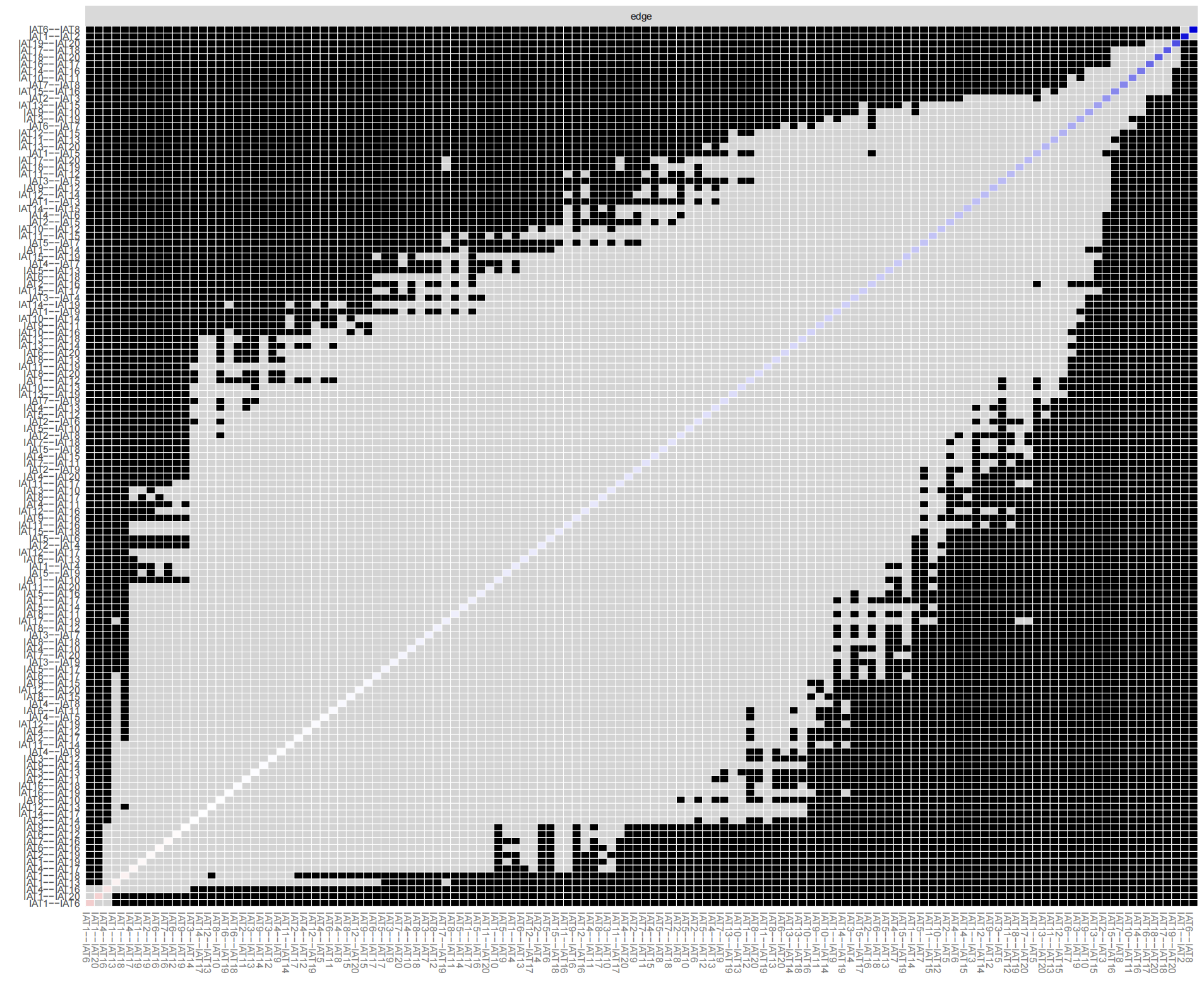


**Supplementary Figure 4. Estimation of edge weight difference by bootstrapped difference test**

Note: Bootstrapped difference tests between edge weights in the network. Gray boxes indicate edges that do not significantly differ from one-another. Black boxes represent edges with significant differences from one another (α = 0.05). Blue boxes in the edge-weight plot indicate positive correlations.
